# Integrated quantitative proteomics and phosphoproteomics analysis reveals USP46-POU4F1-HPSE signaling axis in the pathogenesis of Hirschsprung disease: USP46-POU4F1-HPSE signaling axis in Hirschsprung’s disease

**DOI:** 10.3724/abbs.2025064

**Published:** 2025-04-18

**Authors:** Guowei Li, Fengyin Sun, Jiawei Chen, Qiongqian Xu, Xintao Zhang, Luqiu Chen, Peimin Hou, Aiwu Li

**Affiliations:** Department of Pediatric Surgery Qilu Hospital of Shandong University Jinan 250012 China

**Keywords:** Hirschsprung’s disease, proteomics, USP46, POU4F1, HPSE

## Abstract

Hirschsprung’s disease (HSCR) is a congenital disorder characterized by the absence of enteric ganglion cells in the distal colon, resulting in functional intestinal obstruction. While genetic mutations and microenvironmental imbalances have been implicated in HSCR, the underlying molecular mechanisms are not fully understood. This study uses integrated quantitative proteomics and phosphoproteomics analyses to characterize the differential protein profiles and phosphorylation modifications associated with HSCR. These findings reveal significant dysregulation of the extracellular matrix (ECM) remodelling pathway, suggesting its potential involvement in HSCR pathogenesis. Notably, the deubiquitinating enzyme USP46 is found to be significantly reduced in the aganglionic segments of HSCR patients. Through IP-MS, GST pull-down, and co-immunoprecipitation assays, it is demonstrated that USP46 interacts with the transcription factor POU4F1. Mechanistically, USP46 stabilizes POU4F1 via deubiquitination, increasing its binding to the heparanase (HPSE) promoter and increasing HPSE expression, which in turn promotes ECM remodelling and neural cell migration. The role of the USP46-POU4F1-HPSE signaling axis in HSCR pathogenesis is confirmed via chromatin immunoprecipitation-qPCR, luciferase reporter assays, and transwell migration assays. This study elucidates a novel regulatory mechanism linking USP46-mediated protein stabilization to ECM dynamics and neural cell migration, offering new insights into HSCR pathogenesis and potential therapeutic targets.

## Introduction

Hirschsprung’s disease (HSCR) is a congenital disorder characterized by the absence of ganglion cells in the distal intestine, resulting in functional bowel obstruction, chronic constipation, and Hirschsprung-associated enterocolitis (HAEC) [1]. It occurs in approximately 1 out of every 5000 live births, with males being four times more likely to be affected than females are, suggesting possible sex-linked genetic factors [2]. Even with surgical intervention through procedures such as Swenson, Soave, or Duhamel pull-through, up to half of patients may experience post-surgical issues such as ongoing constipation, enterocolitis, and fecal incontinence [ 3, 4] . This highlights the need for a deeper understanding of its pathophysiology, which involves complex interactions of various cellular and molecular components within the colonic segments.

HSCR is a multifactorial disorder with significant genetic components. The proper function of the enteric nervous system (ENS) depends on the interactions between neurons and glial cells. Originating from neural crest cells (NCCs), which migrate and differentiate into enteric ganglia during embryogenesis, ENS development can be disrupted by genetic mutations, including those in the RET proto-oncogene, EDNRB, GDNF, and SOX10 [ 5, 6] These mutations disrupt NCC migration, proliferation, or differentiation, leading to HSCR. Recent studies have identified new candidate genes, such as ATP7A, SREBF1, ABCD1, and PIAS2, expanding the known genetic landscape of HSCR [6]. The RET/GDNF signaling pathway is crucial for enteric neural crest cell (ENCC) migration, survival, and differentiation [7]. However, known genetic mutations account for only approximately 20% of sporadic HSCR cases, indicating that additional regulatory mechanisms are involved [8]. Even among patients with HSCR-associated mutations, high phenotypic variability suggests that non-genetic factors such as epigenetic regulation and posttranslational modifications contribute to the disease [ 9, 10] . These findings underscore the need to investigate posttranslational regulators involved in ENS development.

The extracellular matrix (ECM) is a critical regulator of ENS progenitor migration, providing both structural support and biochemical signaling [ 11, 12] . In the context of ENS development, several ECM components play key roles: collagen VI (COL6A3) and fibronectin (FN1) regulate the adhesion and differentiation of enteric neural crest cells (ENCCs) [13], and heparanase (HPSE), a key ECM-degrading enzyme, modulates ECM turnover and enhances neural crest cell migration [14]. In HSCR, dysregulated ECM remodelling results in excessive ECM deposition. This accumulation impairs neural crest cell migration and contributes to the failure of ENS development [ 15, 16] . Our previous research demonstrated altered expression levels of collagens I, III, and IV in the colon segments of HSCR patients, changes that may compromise the structural integrity and function of the ENS [17]. However, the molecular mechanisms that connect ECM regulation with HSCR pathogenesis still require further investigation.

Previous studies have extensively explored the genetic and molecular underpinnings of HSCR, revealing the roles of various genes and proteins in the pathogenesis of this condition. However, while there is a wealth of research on these molecular aspects, there has been limited focus on proteomic and phosphoproteomic changes within the affected tissues of HSCR patients. Proteomics and phosphoproteomics, particularly mass spectrometry (MS)-based approaches, provide powerful tools for revealing protein expression profiles and posttranslational modifications that contribute to disease development. Phosphorylation and ubiquitination have emerged as key regulators of ENS development. These modifications influence protein stability (via ubiquitination and deubiquitination), transcriptional regulation (by controlling transcription factor turnover), and neural crest migration (through kinase-mediated phosphorylation cascades) [18].

In the present study, we used MS-based proteomics and phosphoproteomics to identify differentially expressed proteins and phosphorylation events in the aganglionic and normal colon segments of HSCR patients. Our findings provide new insights into the molecular changes in these tissues and offer a comprehensive view of the role of the proteome in the physiopathology of HSCR. Our analyses revealed that USP46 is significantly downregulated in aganglionic HSCR segments, disrupting key pathways associated with ENS migration and ECM remodelling. USP46, a deubiquitinating enzyme (DUB), has been linked to neuronal differentiation and synaptic plasticity [19], where it stabilizes transcription factors essential for neurodevelopment by removing ubiquitin chains from target proteins. However, its role in HSCR pathology has not been explored. On the basis of our findings, we propose that USP46 stabilizes POU4F1, thereby promoting HPSE-mediated ECM remodelling and facilitating neural cell migration. The loss of USP46 disrupts this pathway, leading to reduced HPSE expression, ECM accumulation, and impaired ENS migration, ultimately contributing to HSCR pathogenesis.

## Materials and Methods

### Patient samples and ethics statement

Intestinal tissue samples were collected from HSCR patients undergoing surgery at Qilu Hospital in Jinan, China. The samples were divided into three groups: the aganglionic segment ( *n* = 4), the normal segment ( *n*  = 4), and the mega-colon ( *n*  = 4). After collection, the tissues were immediately frozen in liquid nitrogen and stored at –80°C for subsequent analysis. The study was approved by the Institutional Ethics Committee of Qilu Hospital (approval No: QLU-2024-HSCR-007), and written informed consent was obtained from the legal guardians of all participants.

### Protein extraction and digestion

The tissue samples were collected after washing with PBS three times and were subsequently lysed in SDS lysis buffer (4% SDS, 100 mM Tris-HCl, 0.1 M DTT, pH 7.6). The protein was extracted via sonication (JY92-IIDN; Ningbo Scientz Biotechnology Co., Ltd., Ningbo, China) at 15% amplitude for 5 s on and 5 s off with a total working time of 2 min. The proteins were then denatured and reduced at 95°C for 10 min. The insoluble debris was removed via centrifugation at 12,000 *g* for 15 min, and the supernatant was retained for proteomic experiments. The protein concentration was determined via fluorescence emission at 350 nm with an excitation wavelength of 295 nm. The measurements were performed in 8 M urea using tryptophan as the standard.

The samples were subjected to in-solution trypsin digestion. Briefly, 1 mg of protein was alkylated with iodoacetamide (IAA) in the dark (~200 μL total volume). The protein was precipitated with 1 mL of precooled buffer containing 50% acetone, 50% ethanol, and 0.1% acetic acid and incubated at –20°C overnight. The mixture was subsequently centrifuged at 18,000 *g* for 40 min at 4°C. The protein precipitate was washed with 100% acetone and then 70% ethanol with centrifugation at 18,000 g at 4°C for 40 min. Next, 1 mL of 100 mM NH _4_HCO _3_ was added to each sample and digested with trypsin (Promega, Madison, USA) at a ratio of 1:50 twice for 16 h at 37°C. The resulting peptide mixture was acidified with trifluoroacetic acid (TFA) at a final concentration of 1% and then desalted with a Sep-Pak® tC18 1 cc Vac Cartridge (50 mg; Waters, Milford, USA)) following the manufacturer′s instructions. The amount of the final peptides was determined with a NanoDrop One (Thermo Fisher Scientific, Waltham, USA). Finally, ~500 μg of peptide was collected from each sample. For each sample, 200 ng peptides were used for DIA-based proteomic analysis, and 500 μg peptides were used to enrich phosphopeptides.

### Enrichment of phosphopeptides

Phosphopeptide enrichment was performed via a HighSelect™ Fe-NTA kit (Thermo Scientific Scientific). Briefly, the resins of one spin column were washed with binding/washing buffer (80% ACN and 0.1% TFA) three times and divided into eight aliquots. One aliquot of Fe-NTA was incubated with 500 μg of peptide in 300 μL of binding buffer for 30 min at room temperature, after which the mixture was transferred to a filter tip (TF-20-L-R-S; Axygen, Corning, USA). The supernatant was then removed by centrifugation at 50–200 *g* for 1 min. The resins were washed sequentially with 200 μL of washing buffer three times and 200 μL of H _2_O twice to remove the nonspecifically absorbed peptides. The enriched phosphopeptides were eluted from the resins with 200 μL of elution buffer (50% ACN and 5% NH _3_·H _2_O) twice. The eluted peptides were subjected to vacuum freeze drying and further desalted via C18 StageTips. Half of the purified phosphopeptides were resolved in 0.1% formic acid (FA) and analyzed via LC-MS/MS.

### LC-MS/MS analysis

For both proteomic and phosphoproteomic analysis, the DIA-based method was used for relative proteome quantification. The proteomic data were collected on a timsTOF Pro mass spectrometer (Bruker, Billerica, USA) coupled to a NanoElute ultrahigh-performance liquid chromatography system (Bruker). Tissue peptides (~200 ng) were separated via a 60-min gradient at 400 nL/min via a custom-made microtip column (75 μm ×200 mm) packed with ReproSil-Pur C18-AQ and C18 1.9 μm resin. Mobile phases A and B were water with 0.1% formic acid and acetonitrile with 0.1% formic acid, respectively. A capillary ion source (ion source voltage, 1.5 kV) coupled the NanoElute and mass spectrometer, and the MS data were acquired via the diaPASEF method. The collision energy was ramped linearly as a function of ion mobility from 59 eV at 1/K0 = 1.6 Vs/cm ^2^ to 20 eV at 1/K0 = 0.6 Vs/cm ^2^. The ion mobility was scanned from 0.59 to 1.6 Vs/cm ^2^ with a 100 ms ramp time. Both the full-scan and MS/MS data ranged from 100 to 1700 m/z. The isolation windows were set at 40 m/z in the mass range of 400--1000 m/z in diaPASEF.

For phosphoproteomic analysis, the DIA-based method was used for relative phosphosite quantification on a Q Exactive HF-X mass spectrometer (Thermo Fisher Scientific). The LC gradient was set as follows: 2%–22% B in 72 min; 22%–28% B in 10 min; 28%–100% B in 2 min; and 100% B in 6 min. A total of 32 DIA isolation windows with variable widths were used for phosphoproteomic analysis. The isolation windows were set with a 1 Da overlap as follows: 1 loop count of 57 m/z with central m/z at 378.5; 2 loop counts of 27 m/z with central m/z at 419.5, 445.5; 19 loop counts of 17 m/z with central m/z at 466.5, 482.5, 498.5, 514.5, 530.5, 546.5, 562.5, 578.5, 594.5, 610.5, 626.5, 642.5, 658.5, 674.5, 690.5, 706.5, 722.5, 738.5, 754.5; 4 loop counts of 23 m/z with central m/z at 773.5, 795.5, 817.5, 839.5; 2 loop counts of 33 m/z with central m/z at 866.5, 898.5; 2 loop counts of 47 m/z with central m/z at 937.5, 983.5; 1 loop count of 79 m/z with central m/z at 1045.5; and 1 loop count of 417 m/z with central m/z at 1292.5.

### Database searching of MS data

DIA-NN 1.8.1 software was used to analyze the proteomic data, and Spectronaut v18 software was used to analyze the phosphoproteomic data in spectral library-free mode with the default parameters. All DIA runs were directly searched against the Swiss-Prot human protein database (downloaded in June 2021, 20,600 entries). Trypsin/P was set as the enzyme. Carbamidomethyl (C) was set as the fixed modification, and acetyl (protein N-term) and oxidation (M) were set as the variable modifications. For phosphoproteomic analysis, phospho(S/T/Y) was set as the variable modification, and the PTM localization option was enabled. A PTM localization score > 0.75 was used as the criterion for highly reliable class-I phosphosites.

### Cell culture, transfection and reagents

The human HEK293T and SH-SY5Y cell lines, which were sourced from the Cell Bank of the Chinese Academy of Sciences (Shanghai, China), were maintained in DMEM (Gibco, Carlsbad, USA) enriched with 10% fetal bovine serum (FBS; Gibco) and antibiotics comprising 100 μg/mL streptomycin and 100 U/mL penicillin (Gibco). The samples were incubated in a humidified atmosphere of 5% CO _2_ at 37°C. Full-length USP46 was subsequently cloned and inserted into the pCDNA3.0 vector for overexpression. Human USP46 shRNA knockdown and scramble plasmids (TCREG Biotech, Shanghai, China) were transfected into these cells via Lipofectamine 2000 (Life Technologies, Carlsbad, USA) following the protocol specified by the manufacturer. The targeted sequence of USP46 shRNA plasmid was 5′-CGTGGGCATTATATCACTATT-3′, and the non-targeting control sequence of scramble plasmid was 5′-TTCTCCGAACGTGTCACGT-3′.

### Immunoprecipitation and western blot analysis

The immunoprecipitation (IP) assay was performed as previously described [20]. Briefly, HEK293T cells were lysed in IP buffer (50 mM Tris-HCl, 150 mM NaCl, 5 mM EDTA, and 1% NP-40, pH 7.4) containing a protease inhibitor cocktail (1:100; Beyotime, Shanghai, China). The lysates were incubated with an anti-USP46 antibody (1:100; 13502-1-AP; Proteintech, Wuhan, China) and Protein A/G magnetic beads (C-1002; TCREG Biotech) at 4°C for 6 h and then washed three times with IP buffer. The immunoprecipitates were collected via centrifugation and denatured in 1× SDS-PAGE protein loading buffer at 100°C for 10 min. Half of each sample was used for shotgun mass spectrometry to identify USP46-interacting proteins.

For western blot analysis, the immunoprecipitates, inputs, and other cell lysates were separated by SDS-PAGE and transferred to 0.22 μm PVDF membranes (Millipore, Billerica, USA). The membranes were probed with primary antibodies against POU4F1 (1:1000; ab245230; Abcam, Cambridge, UK), GAPDH (1:5000; 60004-1-Ig; Proteintech), and ubiquitin (1:1000; sc-47721; Santa Cruz, Santa Cruz, USA), followed by incubation with horseradish peroxidase-conjugated secondary antibodies (goat anti-mouse IgG, 1:5000; SA00001-1; Proteintech; goat anti-rabbit IgG, 1:5000; SA00001-2; Proteintech). The signals were detected via a Tanon 5200 Imaging System (Tanon, Shanghai, China), and densitometry analysis was performed with ImageJ software (NIH, Bethesda, USA).

### GST pull-down assay

To assess the direct interaction between USP46 and POU4F1, GST pull-down assays were performed as previously described [21]. Briefly, GST, GST-POU4F1, and 6His-POU4F1 fusion proteins were expressed in *Escherichia coli* BL21 (DE3) and purified via Glutathione Sepharose 4B beads and Ni Sepharose (GE Healthcare, Wisconsin, USA). His-tagged USP46 was incubated with GST- or GST-POU4F1-bound beads at 4°C for 4 h. The beads were washed three times with PBS supplemented with 0.1% Triton X-100 and analyzed via western blot analysis with anti-His (1:1000; 66005-1-Ig; Proteintech) and anti-GST (1:1000; 66001-2-Ig; Proteintech) antibodies.

### Chromatin immunoprecipitation (ChIP-qPCR)

To investigate whether POU4F1 binds to the HPSE promoter, ChIP was performed via the Simple ChIP Kit (Cell Signaling, Danvers, USA). DNA-protein complexes were immunoprecipitated via anti-POU4F1 antibodies, and qPCR was performed using HPSE promoter-specific primers (HPSE-F: 5′-GTCTGTGCACGTGTATATAC-3′; HPSE-R: 5′-TAGTCCCTCTTCCAAATGTC-3′). The ChIP efficiency was calculated via the following method: percentage of input = 5% × 2 ^(C[T] input sample – C[T] IP sample)^.

### Dual-luciferase reporter assay

To confirm the transcriptional regulation of HPSE by POU4F1, the HPSE promoter was cloned and inserted into the pGL3-basic luciferase vector (Promega), and dual luciferase activity was performed as previously described [22]. HEK293T cells were co-transfected with HPSE-promoter constructs, pRL-TK, HPSE-promoter mutation and POU4F1 expression plasmids. Luciferase activity was measured via the Dual-Luciferase Reporter Assay System (Promega).

### Immunofluorescence

SH-SY5Y cells were cultured on glass coverslips and transfected with GFP-POU4F1 and USP46-RFP plasmids via Lipo2000. After 24 h, the cells were fixed with 4% paraformaldehyde (PFA) for 15 min at room temperture and permeabilized with 0.2% Triton X-100. Nuclei were stained with DAPI, and fluorescence was visualized via a Zeiss LSM 880 confocal microscope (Wetzlar, Germany).

### Immunohistochemistry (IHC) of HSCR tissue samples

Paraffin-embedded tissue sections were deparaffinized, rehydrated, and subjected to antigen retrieval in sodium citrate buffer (pH 6.0, 95°C, and 20 min). The sections were incubated with anti-USP46 (13502-1-AP; Proteintech), followed by incubation with HRP-conjugated secondary antibodies (RGAR011; Proteintech). The signals were detected via DAB substrate (Vector Labs, Burlingame, USA).

### Wound healing assay

SHSY5Y cells stably transfected with plasmids containing USP46 or USP46 shRNA were seeded in 6-well plates at 5 × 10 ^4^ cells/mL. When the cells reached 90% confluence, the cell monolayer was scratched with 100 mL pipette tips, washed twice with PBS and cultured with fresh medium for an additional 24 h. Images were captured via an inverted microscope (IX51; Olympus, Tokyo, Japan), and the degree of cell migration to the center of the scratch was measured and calculated.

### Transwell migration assay

To assess neuronal cell migration, transwell assays were performed using polycarbonate membrane inserts (Corning, New York, USA) with 8.0 μm pores. SH-SY5Y cells were transfected with USP46 shRNA or overexpression plasmids and seeded in the upper chamber in serum-free DMEM. The lower chamber contained DMEM supplemented with 10% FBS as a chemoattractant. After 24 h of incubation, the migrated cells were stained with crystal violet, imaged and quantified via ImageJ software.

### Functional enrichment analysis (GO, KEGG pathway, and KSEA analyses)

Gene Ontology (GO) and Kyoto Encyclopedia of Genes and Genomes (KEGG) pathway enrichment analyses were conducted to identify the biological functions and pathways enriched with the differentially expressed proteins. GO analysis (biological process, cellular component, and molecular function) was performed via DAVID (v6.8) and Metascape. KEGG pathway enrichment was conducted via KEGG Mapper and visualized via clusterProfiler (R package). The statistical threshold was adjusted *P*  < 0.05 (Benjamini-Hochberg correction). Overrepresented pathways were visualized via dot plots and bar charts, and significantly enriched terms were mapped onto biological networks via Cytoscape (v3.9.0). Kinase profiling was conducted via the KSEA algorithm [23] on the “Wu Kong” platform ( https://www.omicsolution.org/wkomics/main/) [24]. Kinases with *P* values < 0.05, NetworKIN scores > 2, and substrate counts ≥ 5 were predicted to be significantly enriched. The kinase network was visualized via Cytoscape software.

### Transcription factor-binding site (TFBS) prediction

To identify potential transcription factor (TF) binding sites in the HPSE promoter region, we performed *in silico* promoter analysis. Promoter Sequence Extraction: the –2000 to +100 bp region of the HPSE gene promoter was retrieved from the UCSC Genome Browser. TF binding motif scanning: JASPAR (2024 release) and HOMER (hypergeometric optimization of motif enrichment) were used to identify TFBSs. Position weight matrix (PWM) scoring was applied with a threshold of *P*  < 0.01. The highest scoring TF motifs were validated via ChIP-qPCR to confirm POU4F1 occupancy at the HPSE promoter.

### Statistical analysis

The data generated in this study are expressed as the mean ± SEM and were analyzed via GraphPad Prism 8 (GraphPad Software Inc., San Diego, USA). Statistical significance was determined via two-tailed unpaired Student’s *t* test or one-way ANOVA with Tukey’s *post hoc* test. A *P* value of less than 0.05 was considered to indicate a significant difference.

## Results

### Proteomic analysis of HSCR intestinal segments

**Figure 1:**
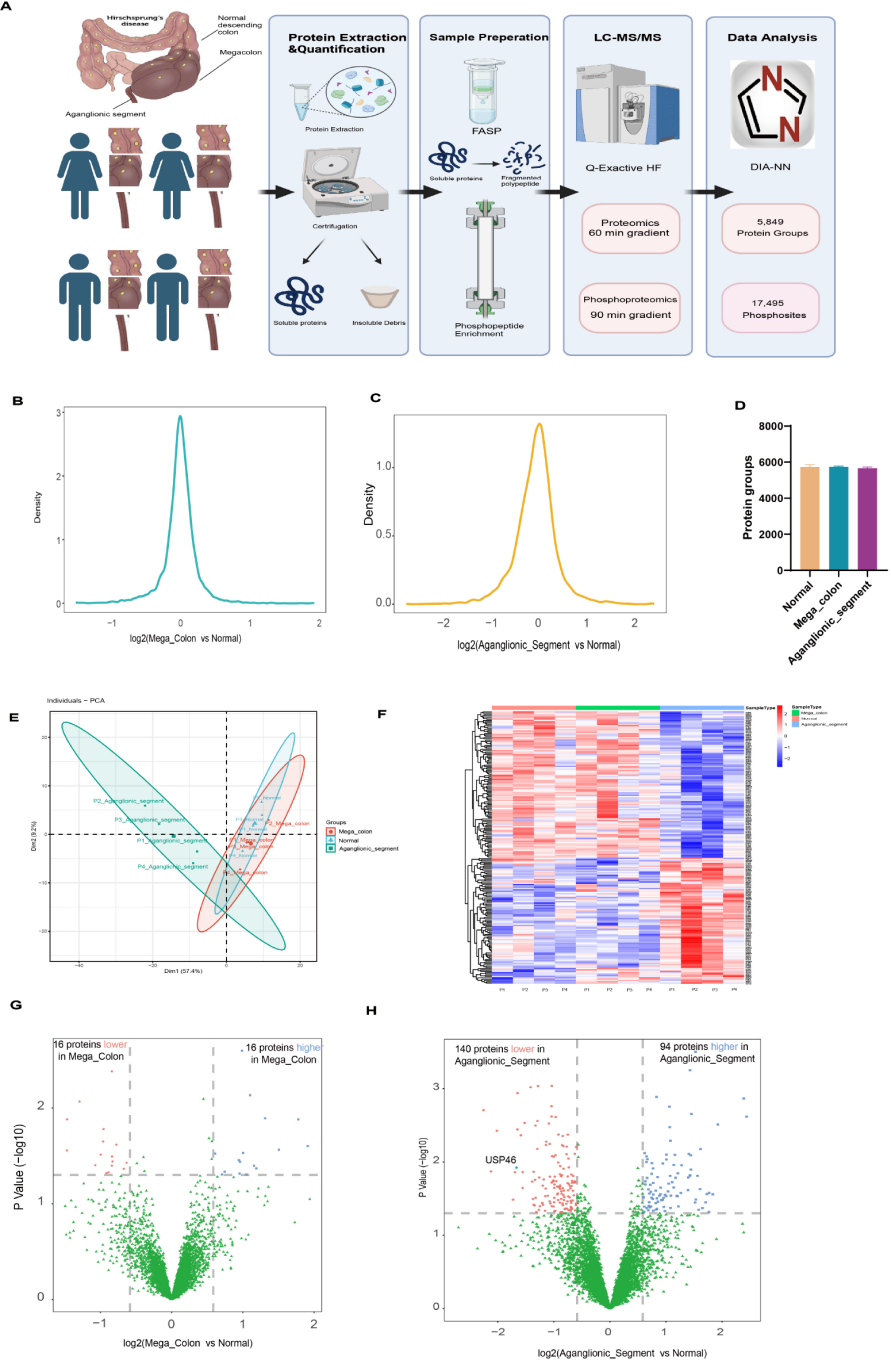
Figure 1 Proteomic workflow and analysis results (A) Schematic representation of the experimental workflow, including protein extraction, sample preparation, LC-MS/MS analysis and data processing steps. (B) Density distribution plots of protein expression changes between megacolon and normal segments, showing that most proteins show subtle variations, with a subset showing significant differential expression. (C) Density distribution plots of protein expression changes between aganglionic and normal segments, showing a broader distribution of differentially expressed proteins. (D) Bar chart showing the number of proteins identified in the three groups (normal, megacolon and aganglionic segments), demonstrating similar protein detection efficiency. (E) Principal component analysis (PCA) plot showing the distribution of samples in the principal component space. The three groups show distinct clustering patterns, indicating clear proteomic differences between normal, megacolon and aganglionic segments. (F) Hierarchical clustering heatmap of differentially expressed proteins (DEPs) across the three groups, showing distinct expression patterns between the normal, megacolon and aganglionic segments. (G) Volcano plots of DEPs of megacolon vs. normal segments, identifying significantly up- and downregulated proteins. (H) Volcano plots of DEPs of aganglionic vs. normal segments, showing a greater number of differentially expressed proteins, reflecting more severe proteomic dysregulation.

To explore the molecular changes associated with HSCR, we conducted label-free quantitative proteomics and phosphoproteomics analyses on aganglionic, megacolon, and normal intestinal segments from four HSCR patients ( Figure 1A). Protein abundance was quantified via label-free quantification (LFQ). Box plots revealed similar distributions of relative LFQ intensities across all samples in aganglionic and megacolon segments ( Figure 1B,C), indicating no significant sample bias. Over 5000 protein groups were identified ( Figure 1D and Supplementary Table S1), yielding a reproducible, high-quality dataset.

Principal component analysis (PCA) revealed limited separation between dilated and normal segments but distinct separation between aganglionic and normal segments ( Figure 1E). This finding suggests similar protein expression in dilated and normal segments, with minor variations, likely due to compensatory mechanisms. In contrast, the aganglionic segment, the disease core lesion area, presented marked differences in protein expression compared with the normal segment, which was consistent with the pathological features of HSCR.

Heatmap clustering analysis corroborated the PCA results ( Figure 1F), highlighting distinct protein expression profiles in the aganglionic, megacolon, and normal segments, with the most significant changes in the aganglionic segment. Volcano plots comparing megacolon segments with respect to normal segments revealed 16 upregulated (blue) and 16 downregulated (red) proteins (fold change > 1.5 or < 0.67, *P*  < 0.05) ( Figure 1G). For the aganglionic segment vs. the normal segment, 94 upregulated (blue) and 140 downregulated (red) proteins were found under the same criteria ( Figure 1H).

**Figure 2:**
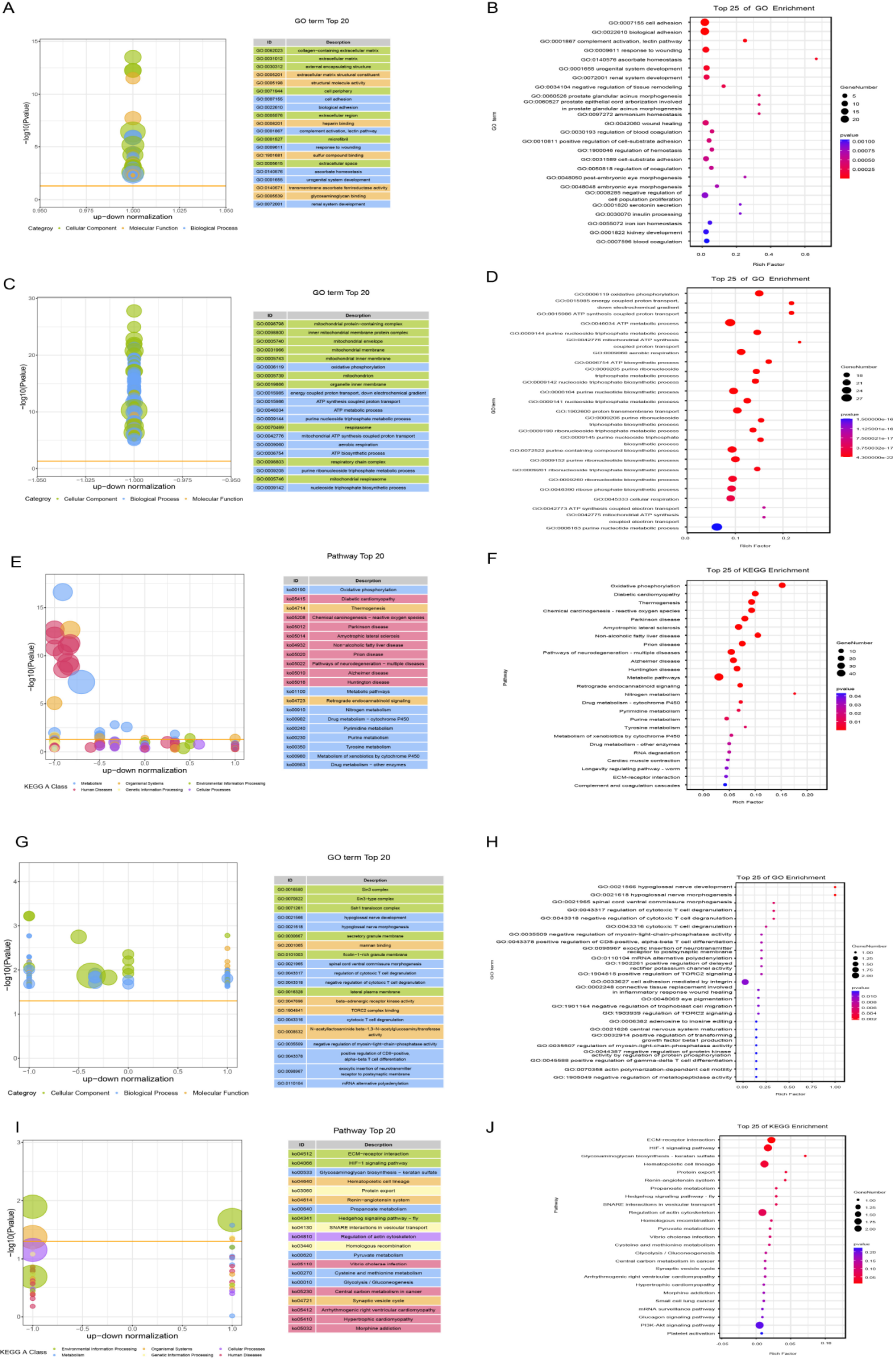
Figure 2 GO and KEGG functional enrichment analyses of proteomic data (A,B) Gene Ontology enrichment analysis of upregulated differentially expressed proteins (DEPs) in aganglionic vs. normal segments. (A) Bubble plot showing the distribution of GO terms, with the x-axis indicating expression trends and the y-axis indicating statistical significance. (B) Bar chart showing the top 25 enriched GO terms for the upregulated proteins. (C,D) GO enrichment analysis of downregulated DEPs in aganglionic vs. normal segments. (C) Bubble plot showing the distribution of GO terms for downregulated proteins. (D) Bar graph showing the top 25 enriched GO terms for downregulated proteins. (E,F) KEGG pathway enrichment analysis of all DEPs in aganglionic vs. normal segments. (E) Bubble plot showing the KEGG pathway distribution. (F) Bar graph highlighting the top 25 enriched KEGG pathways. (G,H) GO enrichment analysis of DEPs in dilated vs. normal segments. (G) Bubble plot showing the distribution of DEPs across GO terms. (H) Bar chart (H) showing the top 25 enriched GO terms for DEPs in expanded segments. (I,J) KEGG pathway enrichment analysis of DEPs in expanded vs normal segments. (I) Bubble plot showing the distribution of DEPs across KEGG pathways. (J) Bar graph showing the top 25 enriched KEGG pathways.

GO analysis revealed that upregulated differentially expressed proteins (DEPs) in aganglionic vs normal segments were enriched in pathways related to cell adhesion, complement activation, and response to injury. The extracellular matrix terms were enriched in the CC and MF categories ( Figure 2A,B). The downregulated DEPs in the aganglionic segment were associated with GO terms related to CC and BP, with mitochondrial function and oxidative phosphorylation being significantly downregulated ( Figure 2C,D).

KEGG pathway enrichment analysis of DEPs between aganglionic and normal segments revealed the following pathways: oxidative phosphorylation, diabetic cardiomyopathy, thermogenesis, chemical carcinogenesis–reactive oxygen species, and Parkinson’s disease ( Figure 2E,F). For DEPs between the dilated and normal segments, GO enrichment highlighted nerve morphogenesis and development ( Figure 2G,H), whereas the enriched KEGG pathways included ECM-receptor interaction, HIF-1 signalling, and glycosaminoglycan biosynthesis-keratan sulfate ( Figure 2I,J).

### Phosphoproteomic analysis of HSCR intestinal segments

**Figure 3:**
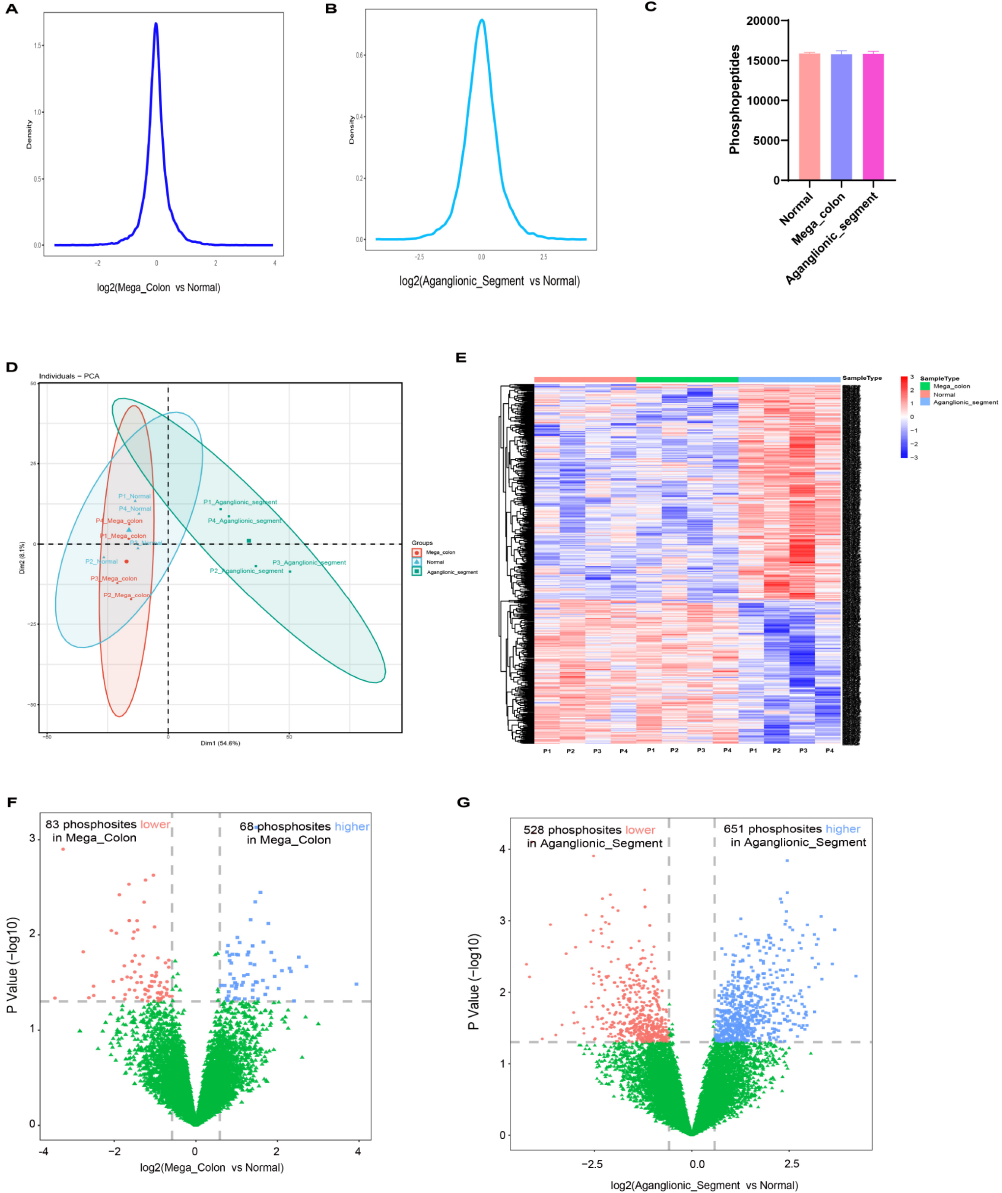
Figure 3 Phosphoproteomic analysis of normal, megacolon and aganglionic segments (A) Density distribution of phosphorylated peptides in megacolon segments vs. normal segments, showing that most phosphorylation changes are concentrated in the central region. (B) Density distribution of phosphorylated peptides in aganglionic vs normal segments, also showing that most phosphorylation changes are concentrated in the central region. (C) Quantification of phosphorylated peptides detected in normal, megacolon and aganglionic segments, showing a comparable number of identified phosphorylation sites in the three groups. (D) Principal component analysis (PCA) plot illustrating the distribution of samples in principal component space, showing a clear separation between the three groups, suggesting systematic differences in phosphorylation patterns. (E) Heatmap of differentially phosphorylated sites showing significant differences in phosphorylation levels between the groups. (F) Volcano plot of differentially phosphorylated sites in megacolon segments vs. normal segments, identifying 83 lower and 68 higher phosphorylation sites. (G) Volcano plot of differentially phosphorylated sites in aganglionic vs. normal segments, showing 528 lower and 651 higher phosphorylation sites, indicating extensive phosphorylation changes in aganglionic HSCR tissues.

Global phosphosite differences across various segments were analyzed ( Figure 3). The relative quantification intensity distributions were consistent among all the samples in both the aganglionic and megacolon segments ( Figure 3A,B), indicating that there was no significant sample bias. Over 15,000 phosphosites were identified across all the samples ( Figure 3C and Supplementary Table S2), establishing a reproducible and high-quality dataset.

PCA and heatmap analysis revealed limited separation between dilated and normal segments, whereas aganglionic segments exhibited distinct separation from normal segments ( Figure 2D,E). Under filtering conditions ( *P*  < 0.05 and |log2 fold change| > 1), phosphoproteomic analysis revealed that, compared with normal segments, giant colon segments presented 83 significantly downregulated and 68 significantly upregulated phosphosites ( Figure 2F). Most changes were small, indicating mild phosphoproteomic differences between these segments. In contrast, aganglionic segments presented 528 downregulated and 651 upregulated phosphosites under the same conditions ( Figure 2G).

GO analysis suggested that upregulated phosphorylated proteins might play crucial roles in RNA metabolism, gene regulation, and nuclear structural maintenance in aganglionic segments ( Supplementary Figure S1A–D,G,H). KEGG pathway enrichment analysis of the phosphoproteomic data revealed significantly enriched pathways related to splicing, cell junctions, signal transduction, metabolism, and disease. These proteins may influence pathological differences between aganglionic and normal segments by regulating signal transmission and metabolic processes, potentially through HSCR mechanisms ( Supplementary Figure S1E,F,I,J).

**Figure 4:**
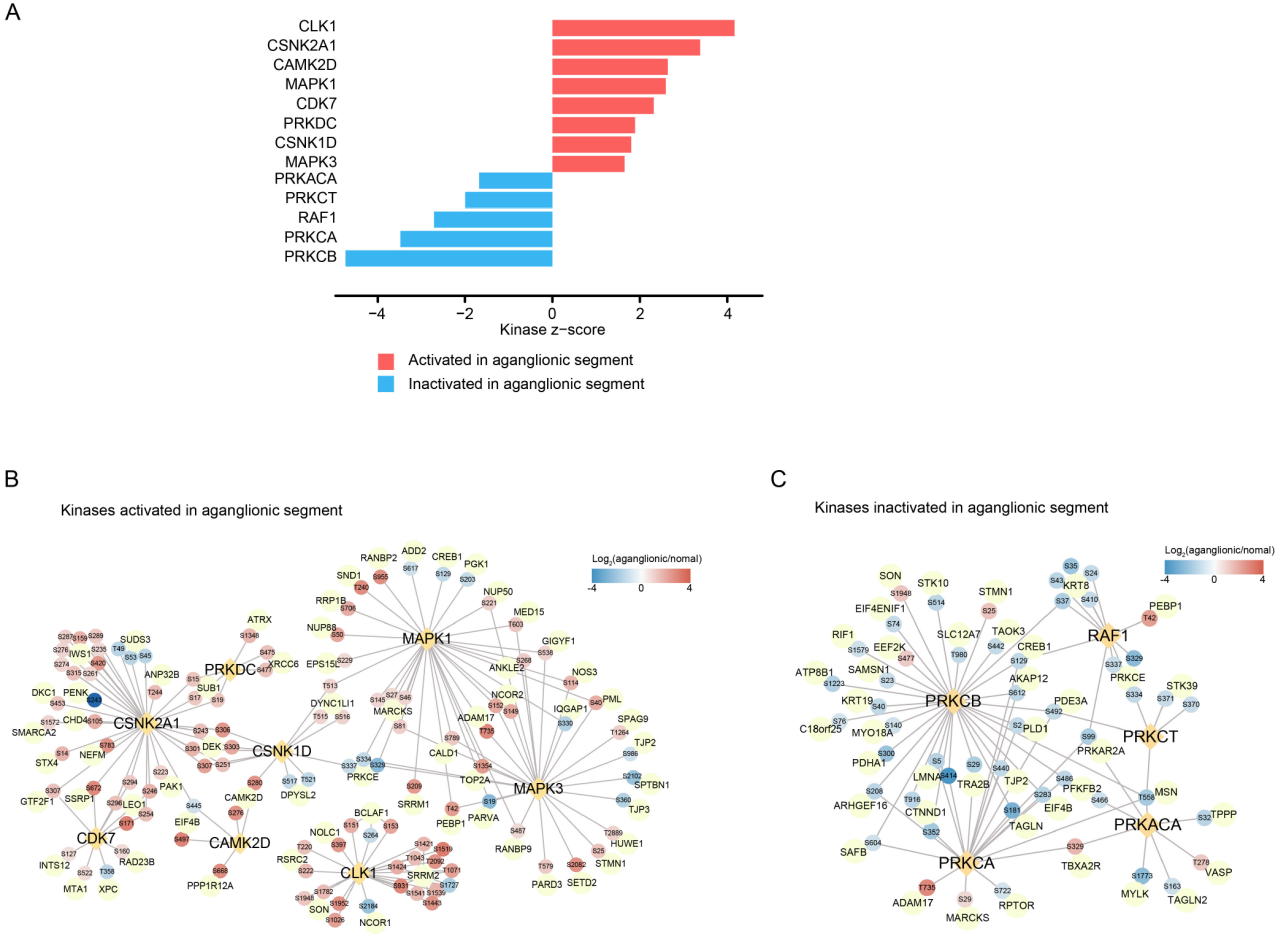
Figure 4 Kinase profiling comparing protein phosphorylation data between aganglionic and normal samples (A) Kinase profiling comparing protein phosphorylation data between aganglionic and normal samples. The kinases shown were selected via the KSEA algorithm on the basis of a P value < 0.05, a NetworKIN score > 2 and ≥ 5 substrates. (B,C) Kinase network describing the interactions of activated (B) and inactivated (C) kinases and their substrates. Yellow diamonds represent kinases, while yellow circles represent substrates. Red circles indicate upregulated phosphorites, and blue circles indicate downregulated phosphorites in aganglionic segment samples.

To explore phosphorylation site regulatory mechanisms, we focused on kinases as key regulatory factors. Using the criteria of a *P* value < 0.05, a NetworKIN score > 2, and at least five substrates, we predicted significantly enriched kinases. Our analysis revealed that CLK1, CSNK2A1, CAMK2D, MAPK1, CDK7, PRKDC, CSNK1D, and MAPK3 were activated in aganglionic segments, whereas PRKACA, PRKCT, RAF1, PRKCA, and PRKCB were inactivated. These findings guided subsequent kinase-related correlation analysis ( Figure 4A). A network diagram illustrates interactions between activated/inactivated kinases and their predicted substrates in aganglionic segments ( Figure 4B,C), revealing key kinases and substrates potentially crucial in aganglionic segment pathogenesis. Understanding these regulatory interactions offers valuable insights into disrupted signaling pathways, paving the way for investigating targeted therapeutic strategies.

### USP46 is downregulated in HSCR aganglionic segments

By integrating proteomic and phosphoproteomic correlation results and conducting enrichment analysis, the probability density plot of the top 20 most enriched functional categories offers key insights into the underlying biological mechanisms ( Supplementary Figure S2A). Enrichment analysis was performed separately using DEGs from the proteome and phosphoproteome, and the oxidative phosphorylation and protein polymerization clusters were visualized ( Supplementary Figure S2B). Circular heatmap visualization was performed to display the overall proteomic and phosphoproteomic data ( Supplementary Figure S2C).

**Figure 5:**
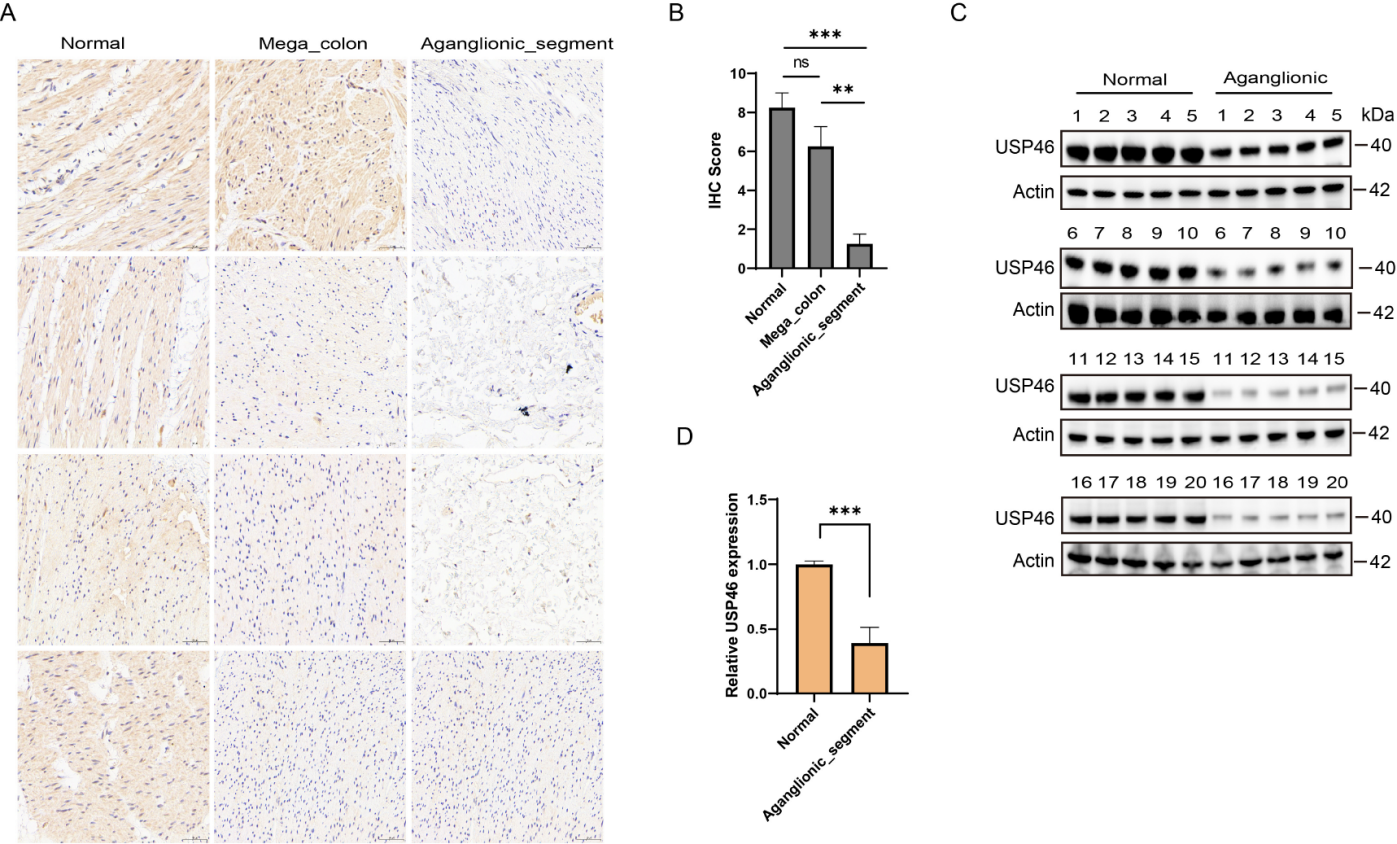
Figure 5 Analysis of USP46 in normal, megacolon and aganglionic segments of HSCR (A) Immunohistochemistry (IHC) staining of normal, megacolon and aganglionic segments showing differential expression of USP46 in different intestinal regions. (B) Quantification of the IHC score shows significantly lower USP46 expression in the aganglionic segment than in the normal segment. (C) Western blot analysis of USP46 protein expression in normal and aganglionic segments, with actin as a loading control. (D) Relative expression levels of USP46 in normal and aganglionic segments. Statistical analysis revealed no significant difference (ns, P > 0.05) between certain groups, whereas other comparisons revealed a significant reduction in USP46 expression (*P < 0.05, **P < 0.01, ***P < 0.001).

Given the high enrichment of K48-linked deubiquitinase activity and that USP46 was the only differentially expressed deubiquitinase in the proteomic dataset, we focused on USP46. Proteomic analysis revealed a 3.2-fold decrease in USP46 levels in HSCR aganglionic segments compared with those in normal segments ( *P*  < 0.001; Figure 1). Western blot analysis and IHC confirmed lower USP46 levels in aganglionic segments. IHC revealed cytoplasmic USP46 localization in normal ganglia, with markedly reduced staining in aganglionic regions ( Figure 5A,B). USP46 expression was further validated via western blotting in fresh HSCR samples, which revealed a 70% reduction in USP46 protein levels in aganglionic tissues ( Figure 5C,D).

### USP46 stabilizes POU4F1 via deubiquitination

**Figure 6:**
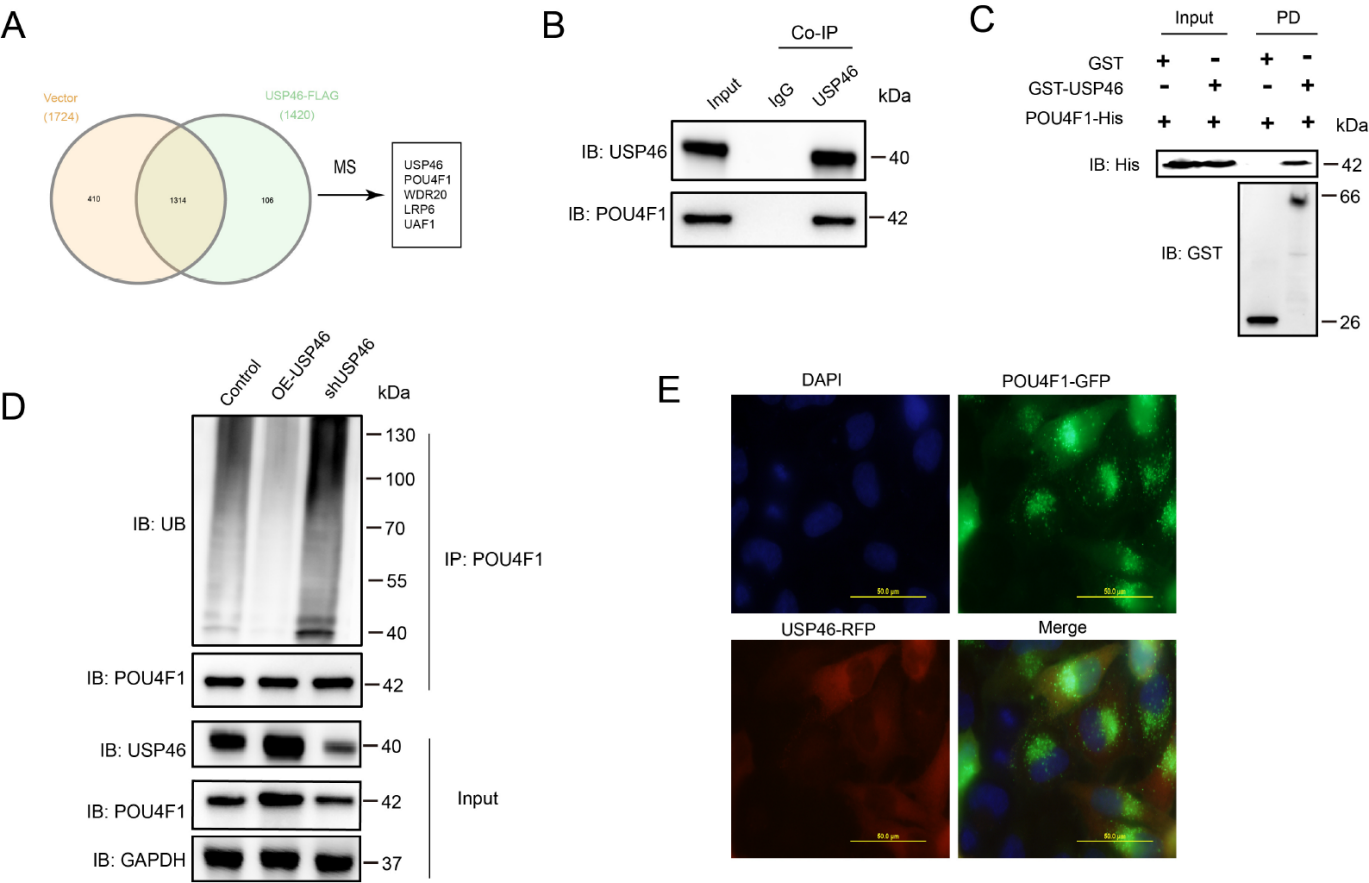
Figure 6 Interaction between USP46 and POU4F1 and its deubiquitination function (A) Immunoprecipitation-mass spectrometry (IP-MS) was used to identify USP46-interacting proteins. The Venn diagram shows the overlap between the USP46 FLAG group (1420 proteins) and the vector control group (1724 proteins), identifying 106 USP46-specific interacting proteins, including POU4F1, WDR20, LRP6 and UAF1. (B) Co-immunoprecipitation (co-IP) confirmed the interaction between USP46 and POU4F1. Immunoprecipitation of USP46 followed by immunoblotting (IB) of POU4F1 revealed that USP46 physically associates with POU4F1. (C) A GST pull-down assay confirmed the direct interaction between USP46 and POU4F1. GST-tagged USP46 successfully pulled down His-tagged POU4F1, whereas the GST control did not, confirming their direct binding. (D) USP46 regulates the ubiquitination status of POU4F1 via deubiquitination. Immunoprecipitation of POU4F1 followed by ubiquitin detection revealed that USP46 overexpression (OE-USP46) significantly reduces POU4F1 ubiquitination, whereas USP46 knockdown (shUSP46) increases its ubiquitination level. (E) Confocal microscopy image showing the subcellular co-localization of USP46 and POU4F1. DAPI stains the nucleus, GFP-tagged POU4F1 is mainly localized in the nucleus, and RFP-tagged USP46 is mainly localized in the cytoplasm. A strong colocalization signal is observed in the perinuclear region, supporting a functional interaction between USP46 and POU4F1. Sacle bar: 50 μm.

Immunoprecipitation coupled with mass spectrometry (IP-MS) was conducted to identify USP46 binding partners in SH-SY5Y cells ( Figure 6A). To assess whether USP46 interacts with POU4F1, coimmunoprecipitation (co-IP) assays and GST pull-down experiments were performed, confirming the direct interaction between USP46 and POU4F1 ( Figure 6B,C).

Further experiments demonstrated that USP46 overexpression reduced POU4F1 ubiquitination, whereas USP46 knockdown increased POU4F1 ubiquitination ( Figure 6D). The ubiquitination level of POU4F1 was not affected by the catalytically inactive C44S mutant of USP46 ( Supplementary Figure S3A). Immunofluorescence staining revealed that nuclear POU4F1 and cytoplasmic USP46 were colocalized near the nuclear membrane ( Figure 6E). These findings indicate that USP46 physically associates with POU4F1 and suggest that USP46 may regulate its stability via deubiquitination.

To test whether USP46 regulates POU4F1 protein stability through its deubiquitinating enzyme activity, ubiquitination assays were performed. The results revealed that USP46 knockdown (shUSP46) significantly increased POU4F1 ubiquitination, leading to POU4F1 degradation ( Figure 6D). Conversely, USP46 overexpression reduced POU4F1 ubiquitination, thereby stabilizing POU4F1 protein levels ( Figure 6D). Collectively, these results demonstrate that USP46 functions as a deubiquitinase for POU4F1, preventing its proteasomal degradation and maintaining its stability in ENS cells.

### Loss of USP46 disrupts POU4F1-dependent transcription programmes

**Figure 7:**
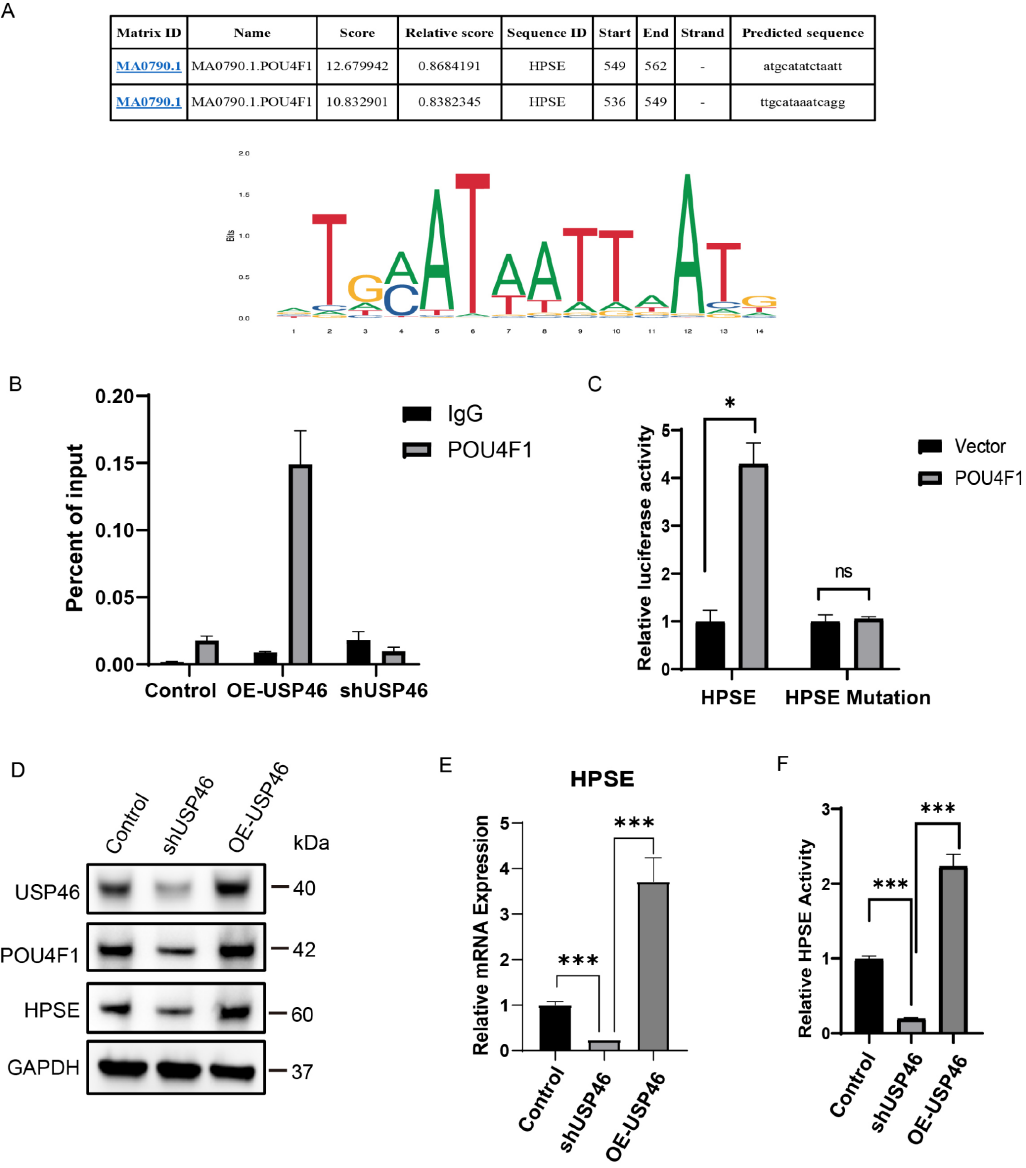
Figure 7 USP46 regulates POU4F1 and its downstream target HPSE (A) JASPAR analysis was used to predict the POU4F1 binding sites in the HPSE promoter and identify the POU4F1 binding motif. (B) ChIP-qPCR revealed that USP46 enhances the DNA binding ability of POU4F1 by stabilizing it. *P < 0.05, ***P < 0.001, n = 3. (C) Dual-luciferase reporter assay results further confirmed that POU4F1 transcriptionally activates HPSE. *P < 0.05, n = 3. (D) Effects of USP46 overexpression or knockdown on the protein levels of POU4F1 and HPSE. (E) A qPCR assay was performed to investigate the effects of USP46 overexpression and knockdown on HPSE mRNA expression. ***P < 0.001, n = 3. (F) Enzymatic activity of HPSE measured under different conditions. ***P < 0.001, n = 3.

POU4F1, a key transcription factor in ENS development, was predicted by JASPAR analysis to have binding motifs in the HPSE promoter ( Figure 7A). ChIP-qPCR revealed that USP46 overexpression increased POU4F1 occupancy at the HPSE promoter 4-fold ( *P*  < 0.001), whereas USP46 knockdown decreased POU4F1 occupancy by 70% ( Figure 7B). Dual-luciferase assays indicated that POU4F1 overexpression significantly enhanced HPSE promoter activity ( *P*  < 0.005; Figure 7C).

To determine whether HPSE is a functional downstream effector of the USP46-POU4F1 axis, we analysed HPSE expression and ECM remodelling under different conditions. In SH-SY5Y cells, USP46 knockdown reduced HPSE mRNA and protein levels by ~50% ( *P*  < 0.01), whereas the reconstitution of the USP46 wild type but not the inactive C44S mutant could upregulate HPSE ( Figure 7D,E and Supplementary Figure S3B). ECM degradation assays revealed that USP46 knockdown decreased HPSE enzymatic activity, mirrored by reduced HS degradation, whereas the USP46 wild type but not the C44S mutant increased HPSE activity ( Figure 7F and Supplementary Figure S3C). This may cause ECM accumulation and impair neural crest cell migration.

### USP46-POU4F1-HPSE axis regulates neural cell migration

**Figure 8:**
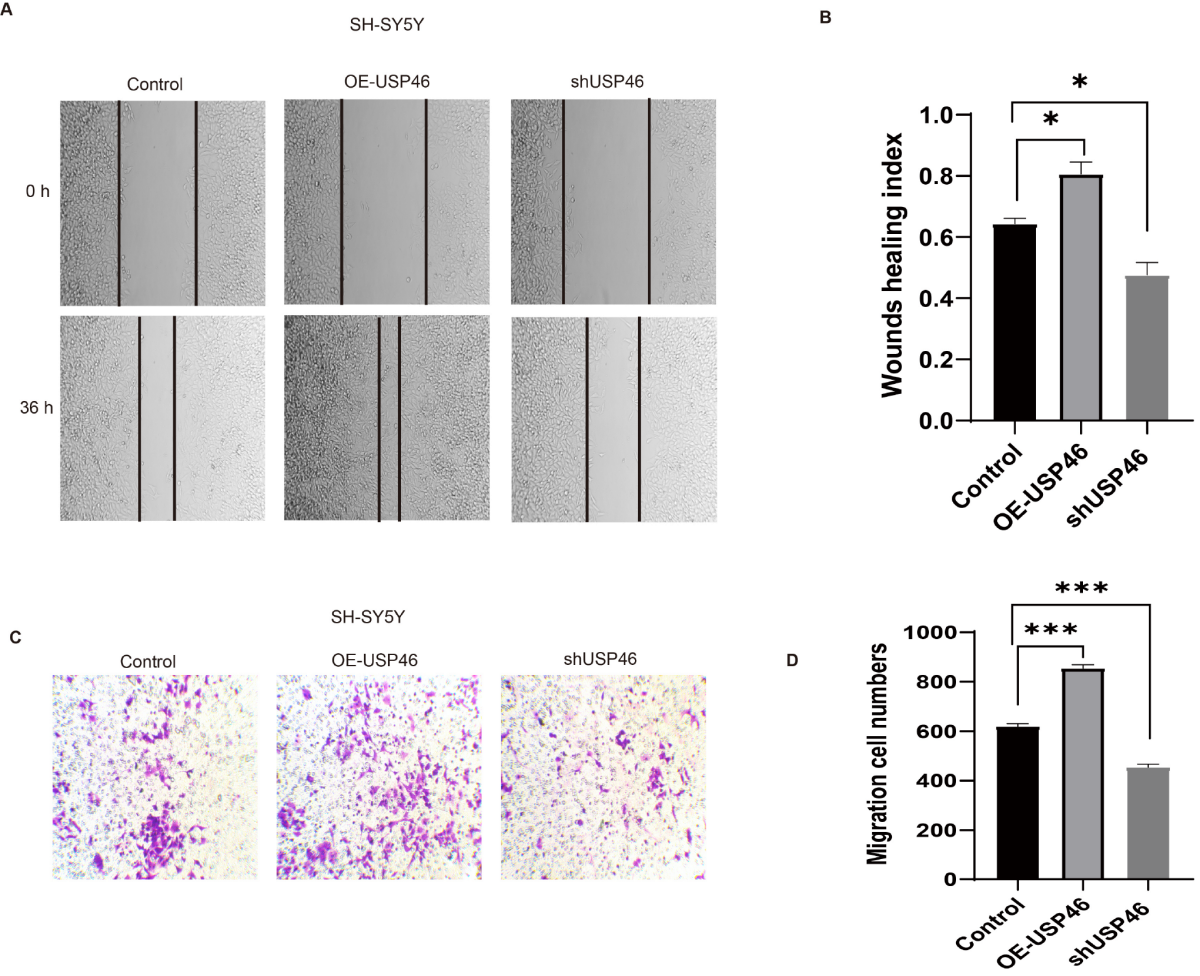
Figure 8 Effect of USP46 on neural cell migration ability (A) Representative images of the wound healing assay at 0 and 36 hours showing wound closure in the control, USP46-overexpressing (OE-USP46) and USP46-knockdown (shUSP46) groups. (B) Quantification of the wound healing index. The results revealed that the healing index was significantly greater in the OE-USP46 group than in the control group, whereas it was significantly lower in the shUSP46 group. *P < 0.05, n = 3. (C) Representative images of the transwell migration assay showing differences in the number of migrated cells among the three groups. (D) Quantification of migrated cells in the transwell assay. The number of migrated cells was significantly greater in the OE-USP46 group, whereas it was significantly lower in the shUSP46 group. ***P < 0.001, n = 3.

To determine whether the USP46-POU4F1-HPSE signaling axis affects ENS migration, we conducted the following experiments. Wound-healing assays revealed an ~45% reduction in SH-SY5Y cell migration upon *USP46* knockdown ( *P*  < 0.01; Figure 8A,B). Transwell migration assays revealed comparable reductions in migratory capacity when USP46 was knocked down ( *P*  < 0.05; Figure 8C,D). These results indicate that the USP46-POU4F1-HPSE axis is crucial for ECM remodelling and ENS migration. USP46 loss reduces HPSE expression, leading to ECM accumulation and defective ENS migration, which contributes to HSCR development.

## Discussion

**Figure 9:**
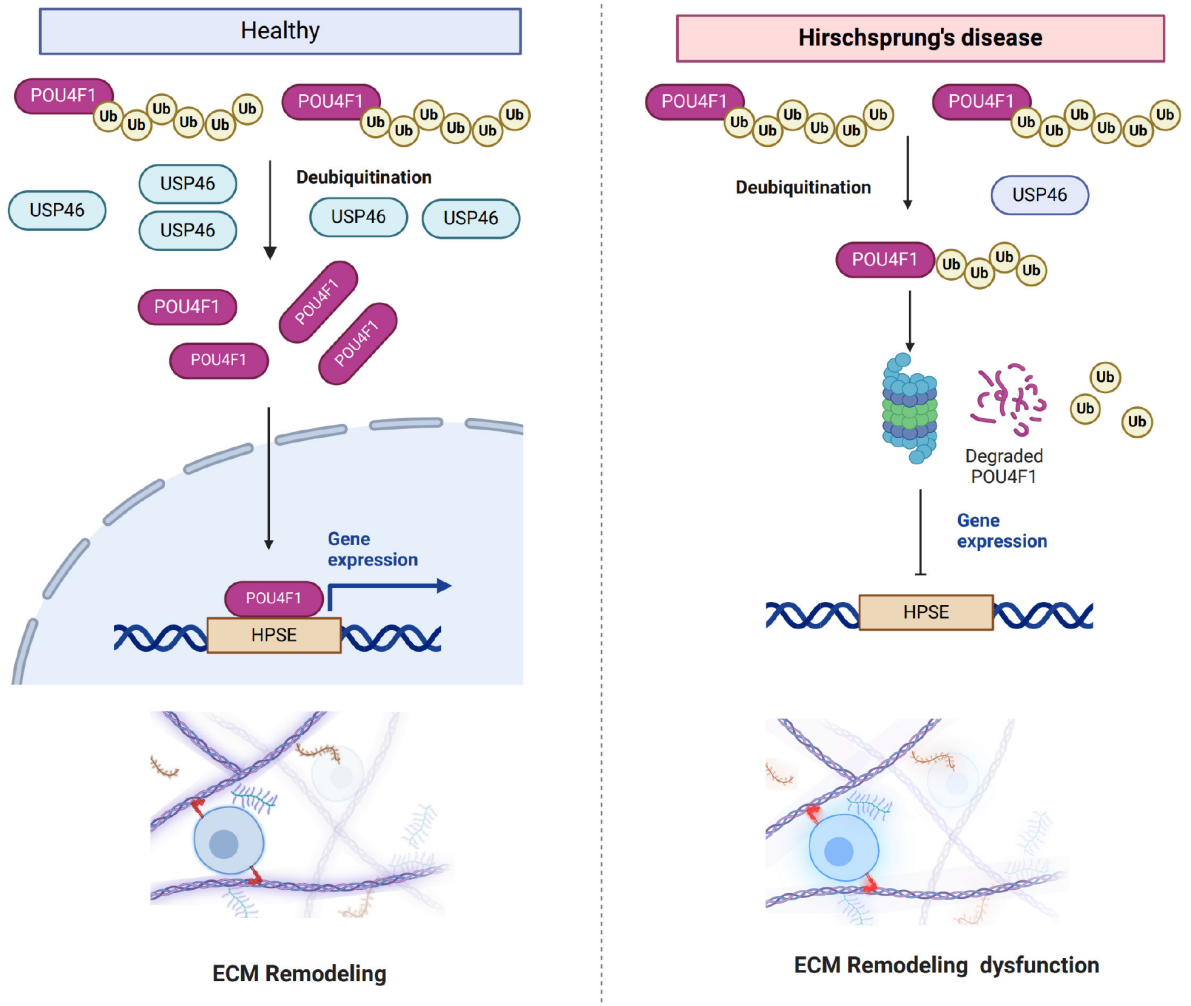
Figure 9 Mechanism of the USP46-POU4F1-HPSE regulatory axis in normal and Hirschsprung′s disease states Under healthy conditions, USP46 stabilizes POU4F1 by deubiquitination, preventing its proteasomal degradation. POU4F1 then binds to the HPSE promoter and activates its transcription. HPSE, a key ECM-degrading enzyme, facilitates the remodelling of the extracellular matrix (ECM) to allow the migration of enteric neurons and the normal development of the enteric nervous system (ENS). Under HSCR conditions, USP46 expression is significantly reduced, leading to increased ubiquitination and degradation of POU4F1. Loss of POU4F1 reduces HPSE transcription, resulting in decreased ECM degradation and excessive ECM accumulation. This inhibits ENS migration and contributes to aganglionosis in HSCR.

Proteomic and phosphoproteomic analyses of HSCR tissues revealed widespread dysregulation of ECM remodelling and neuronal differentiation pathways. USP46 levels are markedly lower in aganglionic HSCR tissues and are associated with reduced migration of enteric neural crest cells (ENCCs) and ECM accumulation. USP46 directly interacts with and deubiquitinates POU4F1, a key transcription factor in ENS development, to prevent its degradation. POU4F1 directly regulates HPSE transcription, linking USP4F1 function to ECM degradation and ENS migration. HPSE likely promotes ENCC migration by cleaving heparan sulfate (HS), which loosens ECM barriers and releases growth factors ( Figure 9). Overall, these findings establish the USP4F1-POU4F1-HPSE axis as a novel regulatory pathway in HSCR pathogenesis, offering potential therapeutic insights.

Ubiquitination is a vital posttranslational modification that significantly impacts numerous biological processes and physiological responses. It directs substrate proteins for degradation, adjusts their activity and function, modifies their subcellular location, and impacts DNA repair [ 20, 25– 28] . Deubiquitination, the reverse of ubiquitination, is executed by the deubiquitinating enzyme (DUB) superfamily [ 29, 30] . USP46, a significant deubiquitinating enzyme, has deubiquitinating activity beyond synaptic regulation, indicating its diverse functions. There are several potential mechanisms and hypotheses for USP46 downregulation in HSCR. First, *USP46* mRNA may be targeted and downregulated by miRNAs, such as miR-33a-5p and miR-27a [ 31, 32] . Second, USP46 might be downregulated due to transcriptional repression or epigenetic silencing ( *e*. *g*., DNA methylation or histone modification) in aganglionic segments. In addition, USP46 might be degraded by the ubiquitin-proteasome system or the autophagy pathway.

Phosphorylation and ubiquitination are two critical post-translational modifications that tightly regulate protein function, localization, stability, and interaction networks. Increasing evidence highlights the dynamic interplay between these modifications, leading to the formation of complex regulatory circuits essential for cellular signaling and homeostasis. In a recent study, CSNK1D was shown to directly phosphorylate USP46 and activate its deubiquitinase activity toward substrates, thus promoting functional changes *in vitro* and *in vivo* [33]. In this study, kinase profiling comparing protein phosphorylation data between aganglionic and normal samples revealed that CSNK1D was activated in aganglionic segments, which may partially rescue USP46 function through increasing deubiquitinase activity.

USP46 ensures sustained HPSE transcription by stabilizing POU4F1, thus associating protein stability with ECM dynamics. This mechanism is similar to USP46’s regulation of AMPA receptors in neurons [19], suggesting its conserved roles in cellular motility.

HPSE, a multifunctional protein with both enzymatic and nonenzymatic functions, plays crucial roles in various biological processes and pathological conditions [34]. The enzymatic and non-enzymatic functions of HPSE may work together to enhance cell migration. In addition to degrading heparan sulfate (HS), HPSE can activate promigratory pathways such as the PI3K/Akt pathway and release ECM-bound cytokines [35]. The HPSE deficiency observed in HSCR may create a rigid ECM microenvironment that hinders enteric neural crest cell (ENCC) colonization [ 15, 36] . Targeting HPSE or USP46 could restore the ECM balance in HSCR. HPSE inhibitors or USP46 activators might increase ENCC migration, suggesting alternatives to invasive surgeries. Our study identified USP46 as a key regulator of ENCC migration via the POU4F1-HPSE axis. Activating USP46 function in HSCR could be a potential therapeutic approach to restoring normal enteric nervous system development. One possible strategy is the use of small molecules that increase USP46 activity or increase its expression. Additionally, post-translational modifications such as phosphorylation or ubiquitination regulators could be explored to increase USP46 stability and activity. Gene therapy approaches, including viral vector-mediated delivery of USP46 or CRISPR activation systems (CRISPRa), can upregulate USP46 expression in enteric neural crest cells, promoting proper neuronal differentiation and migration. Further research is needed to determine the most effective and safe method for activating USP46 in HSCR without disrupting other cellular processes.

While our study revealed novel insights, certain limitations necessitate further research. To validate the *in vivo* role of USP46 in ENS development, future studies should employ USP46 conditional knockout mouse models. Moreover, analyzing larger HSCR patient datasets is essential for evaluating USP46 expression variability and its clinical significance. Another important direction is developing USP46-specific activators for pharmacological modulation.

This study identifies USP46 as a key regulator of ENS development. These findings demonstrate that USP46 stabilizes POU4F1 and transcriptionally activates HPSE, connecting post-translational regulation to ECM remodelling in HSCR pathogenesis. These findings increase our understanding of the molecular mechanisms underlying HSCR and suggest potential therapeutic targets for modulating ENS migration and ECM homeostasis. These findings also open avenues for further exploration of ENS development and congenital intestinal disorders.

## Supporting information

25198Supplementary_table_1

25198Supplementary_table_2

25198supplementary_figures

## Supplementary Data

Supplementary data is available at *Acta Biochimica et Biophysica Sinica* online.
