# Supplementary material for: Integrated quantitative proteomics and phosphoproteomics analysis reveals USP46-POU4F1-HPSE signaling axis in the pathogenesis of Hirschsprung disease: USP46-POU4F1-HPSE signaling axis in Hirschsprung’s disease
Source: Acta Biochim Biophys Sin (Shanghai). 2025 Apr 18;58(2):406–20. doi: 10.3724/abbs.2025064 (PMC12900701; doi:10.3724/abbs.2025064)
Supplement: 25198supplementary_figures [file 25198supplementary_figures.docx]

**
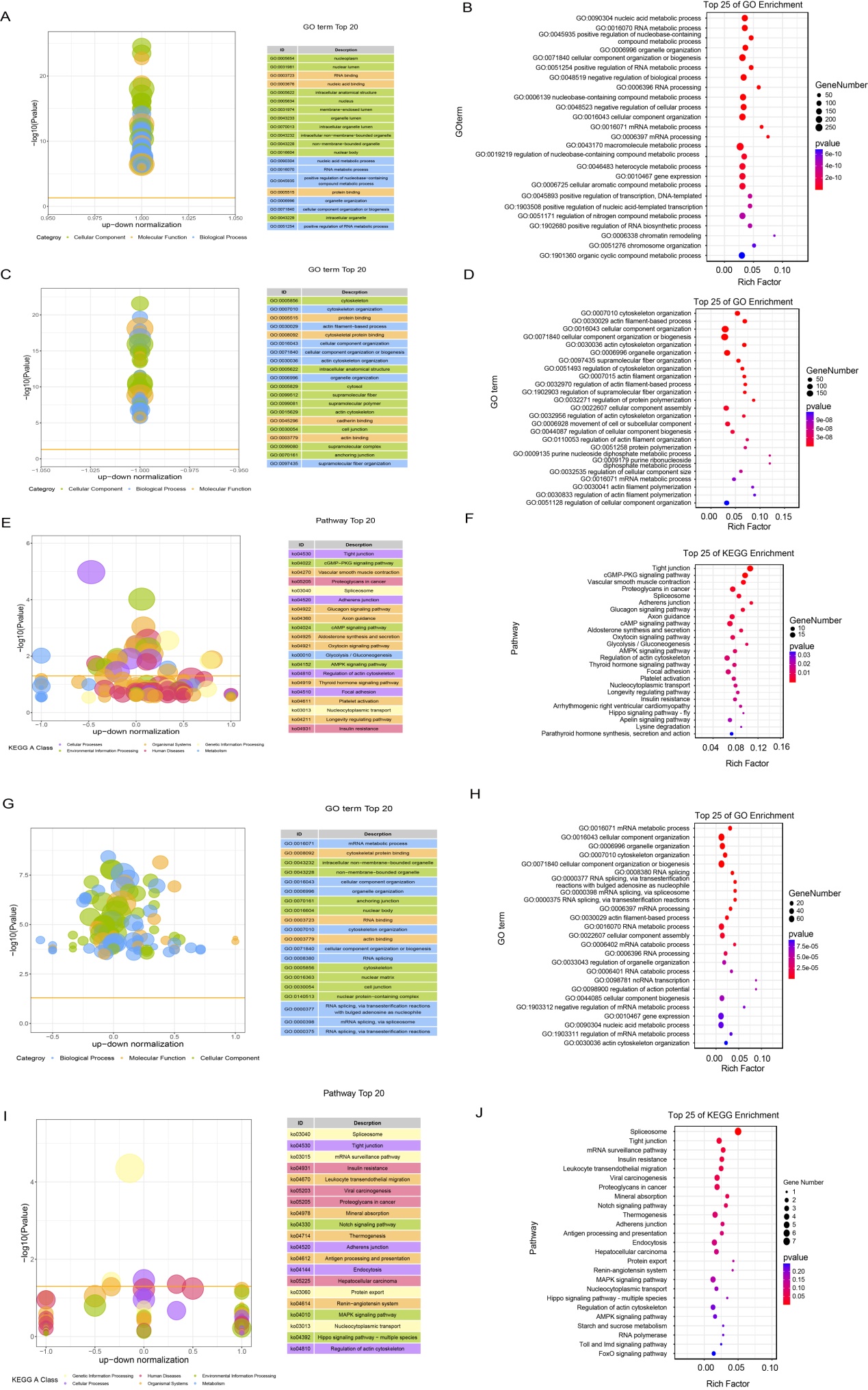
**

**Supplementary Figure S1. GO and KEGG functional enrichment analysis of phosphoproteomics data** (A,B) Gene Ontology enrichment analysis of upregulated differentially expressed proteins (DEPs) in aganglionic vs normal segments. (A) Bubble plot showing the distribution of GO terms, with the x-axis indicating expression trends and the y-axis indicating statistical significance. (B) Bar chart showing the top 25 enriched GO terms for upregulated proteins. (C,D) GO enrichment analysis of downregulated DEPs in aganglionic vs normal segments. (C) Bubble plot showing the distribution of GO terms for downregulated proteins. (D) Bar graph showing the top 25 enriched GO terms for downregulated proteins. (E,F) KEGG pathway enrichment analysis of all DEPs in aganglionic vs normal segments. (E) Bubble plot showing the KEGG pathway distribution. (F) Bar graph highlighting the enriched KEGG pathways. (G,H) GO enrichment analysis of DEPs in aganglionic vs. normal segments. (G) Bubble plot showing the distribution of DEPs across GO terms. (H) Bar chart showing the top 25 enriched GO terms for DEPs in expanded segments. (I,J) KEGG pathway enrichment analysis of DEPs in expanded vs. normal segments. (I) Bubble plot showing the distribution of DEPs across KEGG pathways. (J) Bar chart showing the top 25 enriched KEGG pathways.


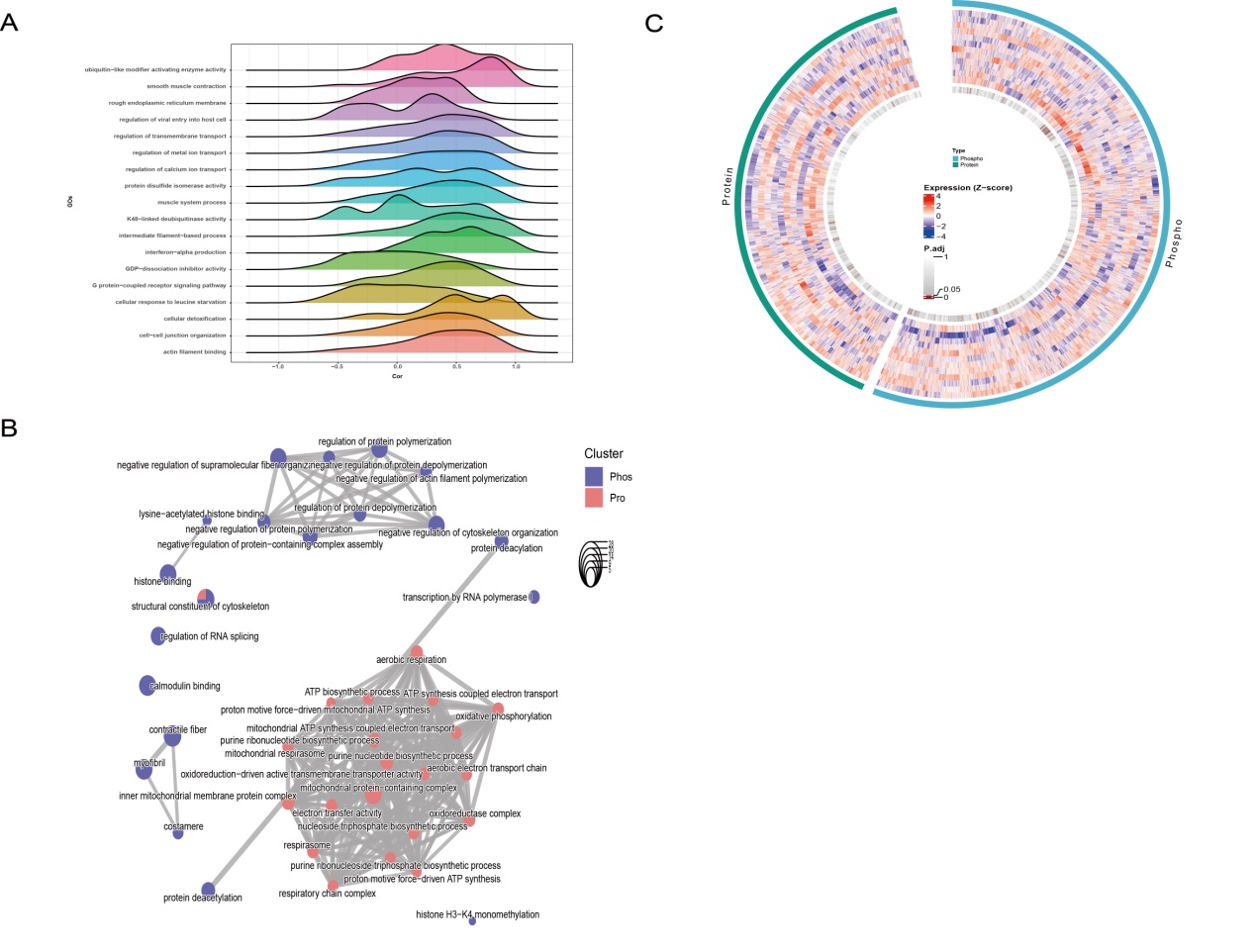


**Supplementary Figure S2.** **Integrated bioinformatics analysis of proteomic and phosphoproteomic datasets**  (A) Gene Ontology (GO) enrichment analysis using genes ranked by correlation strength of proteomic and phosphoproteomic datasets. Top 20 enriched functions were selected based on p-values (Benjamini-Hochberg correction). (B) Enrichment analysis was performed separately using differentially expressed genes (DEGs) from the proteome and phosphoproteome (with a fold change of 2 and *P* < 0.05), and the cluster was visualized. (C) Circular heatmap visualization was performed to display the overall proteomic and phosphoproteomic data. From outer to inner layers: category, quantitative heatmap, fold change, and adjusted *P*-value.

**
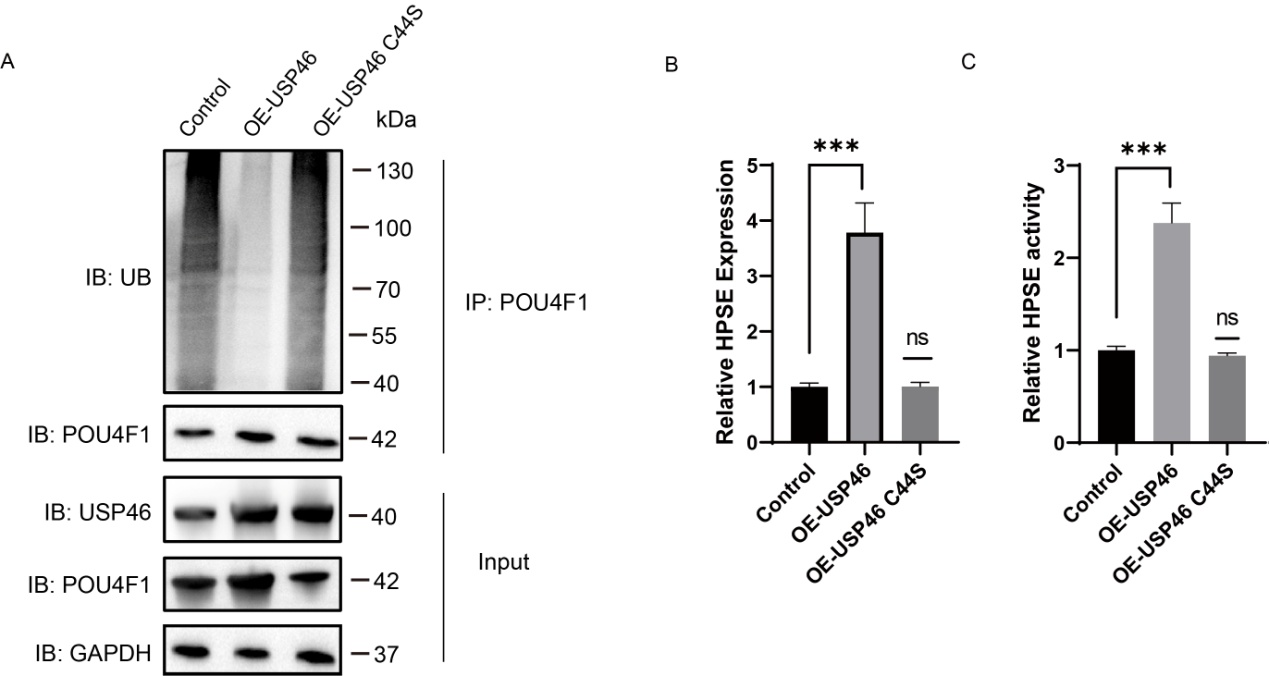
**

**Supplementary Figure S3.** **USP46’s function depends on its deubiquitin** **enzyme activity**  (A) Regulation of POU4F1 ubiquitination depends on USP46 deubiquitination activity. (B) qPCR assay was performed to investigate the effects of USP46 and C44S mutant overexpression on HPSE mRNA expression. ****P* < 0.001, *n* = 3. (C) Enzymatic activity of HPSE measured under different conditions. ****P* < 0.001, *n* = 3.

**Supplementary Table S1. The proteomic dataset**

**Supplementary Table S2. The phosphoproteomic dataset**
